# Hospitalization flow in the public and private systems in the state of Sao Paulo, Brazil

**DOI:** 10.1590/S0034-8910.2015049005696

**Published:** 2015-10-02

**Authors:** Juan Stuardo Yazlle Rocha, Rosane Aparecida Monteiro, Marizélia Leão Moreira

**Affiliations:** I Departamento de Medicina Social. Faculdade de Medicina de Ribeirão Preto. Universidade de São Paulo. Ribeirão Preto, SP, Brasil; II Agência Nacional de Saúde Suplementar. Rio de Janeiro, RJ, Brasil

**Keywords:** Hospitalization, Hospitals, Municipal, supply & distribution, Migration, Regional Health Planning, Equity in Access

## Abstract

**OBJECTIVE:**

To describe the migration flows of demand for public and private hospital care among the health regions of the state of Sao Paulo, Brazil.

**METHODS:**

Study based on a database of hospitalizations in the public and private systems of the state of Sao Paulo, Southeastern Brazil, in 2006. We analyzed data from 17 health regions of the state, considering people hospitalized in their own health region and those who migrated outwards (emigration) or came from other regions (immigration). The index of migration effectiveness of patients from both systems was estimated. The coverage (hospitalization coefficient) was analyzed in relation to the number of inpatient beds per population and the indexes of migration effectiveness.

**RESULTS:**

The index of migration effectiveness applied to the hospital care demand flow allowed characterizing health regions with flow balance, with high emigration of public and private patients, and with high attraction of public and private patients.

**CONCLUSIONS:**

There are differences in hospital care access and opportunities among health regions in the state of Sao Paulo, Brazil.

## INTRODUCTION

According to Viana (2011),^10^ social and health policies in Brazil can be divided into three periods. The period of liberal institutionalism (1995-2002) is characterized by an emphasis on market deregulation and self-regulation with the creation of the National Regulatory Agency for Private Health Insurance and Plans (ANS) and of the Brazilian Health Surveillance Agency (ANVISA), in addition to the key strategy of decentralization with emphasis on primary health care. The transition phase (2003-2006), with positive results in both its balances of trade and payments, developed the emblematic program *Bolsa Família*, a direct money transfer from the state to people in poverty. The neodevelopmentalist institutionalism phase (2007-2010) is characterized by the emergence of the new middle class, which concentrates great purchasing power, and by regionalization in health, combined with the strong expansion of investments geared towards the construction of health facilities – clinics and hospitals – as well as the strengthening of the health care economic and industrial complex.

Studies on the social and economic development have been focusing on the role of the health care sector, which, in addition to the services available, mobilizes the industrial base: chemical, biotechnology and mechanical, electronics and materials. The service sector weighs the most in the health care economic and industrial complex.^2^ Its organization follows spatial organization and configures hubs and regions.

Brazil has only recently started to plan health care in regional levels. Although stated in the 1988 constitutional prescription of the Brazilian Unified Health System (SUS), it advanced only when the *Normas Operacionais de Assistência à Saúde* (NOAS – Operational Health Assistance Regulation) were issued, in 2001 and 2002. The 2002 NOAS spurred the creation of regionalization master plans and the organization of health care networks on regional bases according to the needs of the population, defined by epidemiological parameters – and not by the existence and supply of services.

In 2006, the regionalization and tiering of health services, especially those of high complexity, appear in the political and organizational guidelines from the *Pacto de Gestão* (Management Pact, Brazilian Ministry of Health Ordinance nº 399),^a^ in the regulation and guidelines of the *Pacto pela Vida* and* Pacto de Gestão* (Pact for Life and Management Pact, Brazilian Ministry of Health Ordinance nº 699),^b^ and health care networks.^c^

Oliveira et al^6^ (2008) studied SUS health care in the state of Sao Paulo, focusing on the regional perspective in the context of the design of the State Health Plan. We used data from the *Sistema de Informação Hospitalar* (SIH – Hospital Information System) from 2000 and 2006 for hospital care, and from ANS to estimate the population covered by health plans. In that regard, they state:

“Health managers are at an impasse: to consider or not the coverage of health plans in the SUS planning process? With the growth of the covered population and of the disparity of coverage among regions, particularly among municipalities, not considering this factor may contribute to the maintenance of inequalities in the access currently identified in the system”.

“In the state of Sao Paulo, SUS beds registered in the *Cadastro Nacional de Estabelecimentos de Saúde* (CNES – National Registry of Health Facilities) are mostly philanthropic (46.9% of the beds); 40.2% are public and 12.9% are private beds, under contract with SUS. This feature requires great care in the policy of partnership with the philanthropic hospitals, for fear of jeopardizing care for SUS users, in some regions where there is a higher dependence of these hospital beds”.

Regionalization is questioned by Machado.^4^ Creating health care networks would guarantee fair and comprehensive care to all Brazilian citizens – assuming the articulation between municipalities that “export” and those that “import” patients, under the coordination of the state governments. However, the willingness for cooperation would be far from becoming a reality. Venâncio et al^9^ examined management practices of regional referral in five regions of the state of Sao Paulo, assessing indicators, instruments used and the perception of regional and municipal managers. The formal mechanisms of regional referral were considered insufficient, as well as the instruments for their follow-up.

The regionalization of health has been a concern of health officials for many years,^1^ about the articulation between patient exporter and importer municipalities. The presumed flow logic is that the larger the emigration of patients, the lower the ability to provide care for the population locally or regionally. On the other hand, the higher the immigration of patients, the greater the attraction power exerted by the conditions of care supply. Each region has a hub municipality that concentrates the resources of secondary and tertiary-level health care and would receive the full flow of its referral area. In addition, there are diagnostic and treatment resources of intermediate level in satellite cities of the regional hub. Municipalities and importer regions are also, to some extent, exporters. The predominance of one or another type of flow must be clarified. To this end, it is necessary to consider the size of the referral population, the intensity of the flow in either direction, and the sum of both flows in relation to the total demand in the period.

Studying the hospitalization flow in public and private services can contribute to the knowledge of health needs and demands of the population on regionalized bases, and the adequacy of the structure of the regions and comparisons among them. An objective instrument for evaluating the intensity and direction of the flows is important to the regional structure of health care. The demand for diagnostic and treatment resources outside the place of residence allows inferring the degree of resolution of health care according to the existing care modules. Although the universality principle of SUS establishes the right to health care in any service of the system, it is important to know the origin of patients (municipality of residence) to plan assistance to local and regional demands. Concepts such as import and export, outward or inward referral and patient immigration or emigration are used in the literature with some impropriety.

This study aimed to describe the flow of demand for public and private hospital care among health regions in the state of Sao Paulo, Brazil.

## METHODS

An exploratory study, based on a cross-section of the database built by Moreira^d^ containing the records of all hospitalizations in Brazil by the public and private (health plans and paid by the patient) systems in 2006. Moreira’s study aimed to analyze rehospitalizations in Brazil with data from SIH/SUS and the *Comunicação de Internação Hospitalar* (CIH – Communication of Hospitalizations) system by DATASUS. A database was created by a sophisticated process of record linkage (Reclink) and linkage with the variables name, sex, municipality of residence, and date of birth by means of pre-defined and tested algorithms. As in SIH, each event (hospitalization) may correspond to a partial billing, referring to part of the period or some of the procedures – a situation also accepted in the CIH related to the period –, three algorithms were set to identify the events the same hospitalization and to compose the record of its data. Thus, it was possible to identify the hospitalizations paid by SUS and those paid by the private system as well as their repetitions informed to the Brazilian Ministry of Health by SIH and CIH, respectively.^5^

For this study, we selected all hospitalizations financed by SUS and by the private system of people residing in the state of Sao Paulo, Southeastern Brazil, including the ones in other states of the country, from January 1 to December 31, 2006. The hospitalizations were classified according to the municipality of residence and their location in one of the 17 health regions of the state. The estimated population, the number of beds and beds per 1,000 population of each *Departamento Regional de Saúde* (DRS – Regional Health Department), in 2006, were obtained from the State Health Plan.^8^ Hospitalizations were classified according to each DRS (total hospitalizations) and registered as hospitalizations of patients residing in the same region or not, according to the patient’s city of origin. This enabled estimating the number and percentage of patients assisted in their own region; the total number of hospitalizations in each region (column C, Tables 1 and 2) minus the total number of people hospitalized in their region of origin (column E, Tables 1 and 2), which provides the number of immigration cases, i.e., the number of cases coming from other regions; the total number of hospitalized residents (column D, Tables 1 and 2), minus the total number of people hospitalized in their region of origin (column E, Tables 1 and 2), provides the number of cases of emigration, in which the hospitalization occurred outside the region of the patient. The total number of people hospitalized in each DRS, in relation to the resident population per 1,000 inhabitants, shows the coefficient of total hospitalization of each region of the state.

The intensity of the immigration and emigration of patients was estimated by the index of migration effectiveness (ME), used by Caiado.^2^ This indicator allows quantifying the intensity of patient inflow and outflow and the predominant direction. It is estimated by the net migration rate (immigration less emigration) and the crude migration (immigration plus emigration).


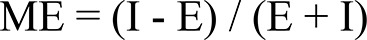


Values close to 1 indicate a strong migratory attraction, close to -1 indicate areas of high emigration, and close to 0, areas with high migratory circulation.

We estimated correlation coefficients between the number of hospital beds and the coefficient of hospitalizations per 1,000 population. We also analyzed the index of migration effectiveness of immigration and emigration o patients from the public (SUS) and private (non-SUS) systems in each Regional Health Department.

## RESULTS

There were 2,922,648 hospitalizations in the state of Sao Paulo in 2006, of which 2,808,765 were of Sao Paulo residents hospitalized in this state and 83,389 hospitalized in other states, totaling 2,892,154 hospitalizations of Sao Paulo residents (Table 3). The hub cities have just over 15 million inhabitants, which means that most of the regions have a greater population in the satellite referral area than residing in the hubs – almost 10 times greater in Taubate and Sao Joao da Boa Vista and a little less than the double in the Greater Sao Paulo. The resulting services demand flows represented the dynamics of regionalization in the state.

**Table 3:** Distribution of population, hospitalizations, index of beds/population, and coefficient of hospitalization, according to health region. State of Sao Paulo, Southeastern Brazil, 2006.

Health Regions	Population	Population in the hub city	% Population in the hub	Total resident hospitalizations	Beds/1,000 population rate	Coefficient of hospitalization per 1,000 inhabitants (residence/population)
3501 Greater Sao Paulo	19,677,510	10,603,309	53.9	1,168,968	1.36	59.41
3502 Aracatuba	700,008	174,770	25.0	55,645	2.12	79.49
3503 Araraquara	915,240	192,153	21.0	74,223	1.52	81.1
3504 Baixada Santista	1,666,453	402,664	24.2	116,692	1.46	70.02
3505 Barretos	412,722	106,048	25.7	43,081	2.55	104.38
3506 Bauru	1,623,025	343,285	21.1	153,389	2.45	94.51
3507 Campinas	3,885,612	1,019,655	26.2	273,450	1.19	70.38
3508 Franca	657,344	315,795	48.0	55,075	1.57	83.78
3509 Marilia	1,081,290	215,673	19.9	111,738	2.36	103.34
3510 Piracicaba	1,405,849	352,684	25.1	85,089	1.31	60.52
3511 Presidente Prudente	723,244	198,937	27.5	80,297	2.41	111.02
3512 Registro	299,360	55,141	18.4	13,462	1.03	44.97
3513 Ribeirao Preto	1,261,413	538,639	42.7	88,713	1.82	70.33
3514 Sao Joao da Boa Vista	797,952	80,334	10.1	83,692	2.05	104.88
3515 Sao Jose do Rio Preto	1,459,320	399,904	27.4	186,250	2.49	127.63
3516 Sorocaba	2,245,623	556,366	24.8	168,920	1.24	75.22
3517 Taubate	2,243,796	261,454	11.6	133,470	1.42	59.48
Total	41,055,761	15,816,811	38.5	2,892,154	1.53	70.44

The global rate of beds per population in the state was low (1.53 per 1,000 population), with wide variation between regions (Table 3). The Regional Health Departments that presented the highest percentage of beds per inhabitants were Barretos (2.6), Sao Jose do Rio Preto (2.5), Bauru (2.5), and Presidente Prudente (2.4). Barretos and Bauru have specialized hospitals that are national references in the areas of Oncology and craniofacial abnormalities, which explains their high number of beds. The Regional Departments with lowest rates of beds per population were Registro (1.0), Campinas (1.2), and Sorocaba (1.2). The global hospitalization coefficient including public and private hospitals (SUS and private system) of the Sao Paulo state was low (70.4 hospitalizations per 1,000 population), although it showed wide variation between regions. The health regions with the greatest coefficients of hospitalizations per 1,000 inhabitants were Sao Jose do Rio Preto (127.6), Presidente Prudente (111.0), Sao Joao da Boa Vista (104.9), Barretos (104.4), and Marilia (103.3). Those with the lowest coefficients of hospitalization of the resident population were Registro (45.0), the Greater Sao Paulo (59.4), Taubate (59.5), and Piracicaba (60.5). The estimation of the correlation coefficient indicated a high positive association between the index of beds per population and the coefficient of hospitalizations per 1,000 population (r = 0.88). In the state average, 69.9% of hospitalizations occurred in SUS and the highest percentage occurred in the regions of Registro (95.8), Aracatuba (86.8), and Taubate (81.0), and the lowest in Presidente Prudente (61.4), Campinas (62.2), and Franca (64.2) (Table 1). In general, the hospitalizations by SUS occurred in the patient’s region in 97.6% of cases – suggesting that the regionalization of SUS is appropriate, i.e., 2.4% sought care successfully in another region (emigration). The health regions provided care for people from other regions of the state (immigration) in 3.2% of the cases. All health regions had cases of immigration and emigration (columns F and G). Estimating migration effectiveness (column H) enabled quantifying and detecting the predominance of the flow in one direction or another. Positive values above 0.50 were considered evidence of strong migratory attraction (immigration) for SUS, which was the case of the regions of Bauru (0.78), Ribeirao Preto (0.69), Barretos (0.65), and Sao Paulo (0.56), i.e., regions that attract patient flows for SUS care. Conversely, the regions that presented negative migration effectiveness indexes (below -0.50) were considered of strong SUS emigration, which was the case for the regions of Franca (-0.80), Marilia, Araraquara and Registro (-0.77), and Aracatuba (-0.71), i.e., regions that stimulate emigration and fail to cover the local demand, although Aracatuba and Marilia have high beds/population rates.

**Table 1:** Total hospitalizations by the Brazilian Unified Health System (SUS) according to the service location, total resident hospitalizations, hospitalizations in the place of origin, immigration and emigration (number and coefficient) and index of migration effectiveness. State of Sao Paulo, Southeastern Brazil, 2006.

Health regions	SUS hospitalizations by service location (C)		Total resident hospitalizations (D)	Hospitalizations in the place of origin (E)		Immigration cases (F)		Emigration cases (G)		Index of migration effectiveness (H)
	n	%		n	%	n	%	n	%	
3501 Greater Sao Paulo	834,367	70.5	821,626	816,707	99.4	17,660	2.1	4,919	0.6	0.56
3502 Aracatuba	44,840	86.8	47,117	44,370	94.2	470	1.0	2,747	5.8	-0.71
3503 Araraquara	47,057	66.5	49,966	46,612	93.3	445	0.9	3,354	6.7	-0.77
3504 Baixada Santista	75,347	66.2	77,121	74,569	96.7	778	1.0	2,552	3.3	-0.53
3505 Barretos	35,205	74.7	29,531	27,992	94.8	7,213	20.5	1,539	5.2	0.65
3506 Bauru	124,326	75.0	112,994	111,427	98.6	12,899	10.4	1,567	1.4	0.78
3507 Campinas	179,028	62.2	174,807	170,262	97.4	8,766	4.9	4,545	2.6	0.32
3508 Franca	34,050	64.2	36,016	33,810	93.9	240	0.7	2,206	6.1	-0.80
3509 Marilia	78,919	72.9	82,005	78,466	95.7	453	0.6	3,539	4.3	-0.77
3510 Piracicaba	59,262	72.0	62,199	58,298	93.7	964	1.6	3,901	6.3	-0.60
3511 Presidente Prudente	49,215	61.4	50,122	48,915	97.6	300	0.6	1,207	2.4	-0.60
3512 Registro	11,729	95.8	12,731	11,579	91.0	150	1.3	1,152	9.0	-0.77
3513 Ribeirao Preto	71,734	76.4	67,247	66,220	98.5	5,514	7.7	1,027	1.5	0.69
3514 Sao Joao da Boa Vista	53,505	66.1	56,639	52,449	92.6	1,056	2.0	4,190	7.4	-0.60
3515 Sao Jose do Rio Preto	113,262	58.3	111,737	109,302	97.8	3,960	3.5	2,435	2.2	0.24
3516 Sorocaba	123,793	74.4	125,766	121,252	96.4	2,541	2.1	4,514	3.6	-0.28
3517 Taubate	106,506	81.0	107,498	104,545	97.3	1,961	1.8	2,953	2.7	-0.20
Total	2,042,145	69.9	2,025,122	1,976,775	97.6	65,370	3.2	48,347	2.4	0.15

In the state average, 30.1% of hospitalizations occurred in private systems, the highest in the regions of Sao Jose do Rio Preto (41.7), Presidente Prudente (38.6), Campinas (37.8), and Franca (35.8); and the smallest in Registro (4.2), Aracatuba (13.2), and Taubate (19.0) (Table 2). The estimation of the index of migration effectiveness indicated the regions of Sao Jose do Rio Preto (0.87) and Campinas (0.64) as those most attracting hospitalizations by private plans. Emigration in search of private assistance was more intense in Registro (-0.87), the region with the lowest beds/population rate, Aracatuba (-0.81), Barretos (-0.70) and Baixada Santista (-0.56).

**Table 2:** Distribution of total hospitalizations financed by private systems, immigration and emigration (number and coefficient) and index of migration effectiveness. State of Sao Paulo, Southeastern Brazil, 2006.

Health regions	Total private system hospitalizations by service location (C)		Total resident hospitalization (D)	Hospitalizations in the place of origin (E)		Immigration cases (F)		Emigration cases (G)		Index of migration effectiveness (H)
	n	%		n	%	n	%	n	%	
3501 Greater Sao Paulo	348,423	29.5	347,342	335,934	96.7	12,489	3.6	11,408	3.3	0.05
3502 Aracatuba	6,815	13.2	8,528	6,615	77.6	200	2.9	1,913	22.4	-0.81
3503 Araraquara	23,690	33.5	24,257	22,405	92.4	1,285	5.4	1,852	7.6	-0.18
3504 Baixada Santista	38,454	33.8	39,571	38,013	96.1	441	1.1	1,558	3.9	-0.56
3505 Barretos	11,923	25.3	13,550	11,571	85.4	352	3.0	1,979	14.6	-0.70
3506 Bauru	41,401	25.0	40,395	38,957	96.4	2,444	5.9	1,438	3.6	0.26
3507 Campinas	108,733	37.8	98,643	95,863	97.2	12,870	11.8	2,780	2.8	0.64
3508 Franca	18,973	35.8	19,059	18,345	96.3	628	3.3	714	3.7	-0.06
3509 Marilia	29,272	27.1	29,733	27,832	93.6	1,440	4.9	1,901	6.4	-0.14
3510 Piracicaba	23,077	28.0	22,890	20,918	91.4	2,159	9.4	1,972	8.6	0.05
3511 Presidente Prudente	30,964	38.6	30,175	29,330	97.2	1,634	5.3	845	2.8	0.32
3512 Registro	519	4.2	731	503	68.8	16	3.1	228	31.2	-0.87
3513 Ribeirao Preto	22,181	23.6	21,466	20,861	97.2	1,320	6.0	605	2.8	0.37
3514 Sao Joao da Boa Vista	27,390	33.9	27,053	25,390	93.9	2,000	7.3	1,663	6.1	0.09
3515 Sao Jose do Rio Preto	81,149	41.7	74,513	74,033	99.4	7,116	8.8	480	0.6	0.87
3516 Sorocaba	42,569	25.6	43,154	41,256	95.6	1,313	3.1	1,898	4.4	-0.18
3517 Taubate	24,970	19.0	25,972	24,164	93.0	806	3.2	1,808	7.0	-0.38
Total	880,503	30.1	867,032	831,990	96.0	48,513	5.5	35,042	4.0	0.16

The study of hospital demand flows in the state of Sao Paulo in 2006 showed as problematic the regions of Aracatuba, Registro and Baixada Santista, which presented strong emigration of patients to public and private care, indicating a lack of both types of health care (Table 4). The regions of Araraquara, Franca, Marilia, Piracicaba, Presidente Prudente, and Sao Joao da Boa Vista showed a high rate of migration for public care. Two regions – Campinas and Sao Jose do Rio Preto – had a high attraction to private care, and both showed balance in the SUS flows. The region of Ribeirao Preto showed high public flow and balance in private flows. The regions of Sorocaba and Taubate showed balance in public and private flows.

**Table 4:** Characterization of the Health Regions of the state of Sao Paulo, according to patient flows for hospital public and private care. State of Sao Paulo, Southeastern Brazil, 2006.

Health Regions	Flow balance		Strong flow attraction		Strong flow emigration	
	Public	Private	Public	Private	Public	Private
Greater Sao Paulo		X	X			
Aracatuba					X	X
Araraquara		X			X	
Baixada Santista					X	X
Barretos			X			X
Bauru		X	X			
Campinas	X			X		
Franca		X			X	
Marilia		X			X	
Piracicaba		X			X	
Presidente Prudente		X			X	
Registro					X	X
Ribeirao Preto		X	X			
Sao Joao da Boa Vista		X			X	
Sao Jose do Rio Preto	X			X		
Sorocaba	X	X				
Taubate	X	X				

## DISCUSSION

The municipalities of the state of Sao Paulo had major differences regarding socioeconomic development and population density in 2009.^7^ Of them, 11.3% had over 100,000 inhabitants and all were classified as high-wealth in the *Índice Paulista de Responsabilidade Social* (Sao Paulo Index of Social Responsibility); 61.7% had less than 20,000 inhabitants and 70.4% were classified as of low wealth. Although only 73 municipalities were included among the large ones, they had nearly 75.0% of the state population. This illustrates the great dependence of the smallest municipalities on the largest ones and, therefore, the importance of the issue of regionalization of health in the state of Sao Paulo. Larger, wealthier municipalities become hubs and attract the population of smaller, poorer municipalities. Thus, regionalization is the instrument to try to compensate for inequality and provide equal opportunities for disadvantaged populations.

The invention of the migration effectiveness indicator, applied to the study of the flows of the demand for hospitalization in the state of Sao Paulo, resulted in an index that allows quantitatively ranking the proportion of cases of patient immigration and emigration among health regions of the state, and the higher prevalence of one or another. This perspective facilitates the qualitative classification of municipalities and health regions according to balance in migration flows or strong attraction or emigration of patients for public (SUS) or private care. It was surprising to find out that the region of Greater Sao Paulo – hub of hospital resources, especially of tertiary level, but with large population concentration – presented a low coefficient of hospitalization of the local population, suggesting a possible relative shortage of beds due to a much higher external demand. The regions of Sao Jose do Rio Preto and Campinas showed no patient emigration for SUS – the first one with high beds/population rate, unlike the second one. Registro showed a high emigration for private hospital care demand, probably for lack of public care supply. Aracatuba, with 2.12 beds per 1,000 inhabitants, presented high emigration for private care, probably for problems of access to existing beds.

While the total number of cases detected as immigration or emigration is relatively small (5.6% and 9.5% of the public and private hospitalizations), it afflicts a population weakened by disease. The aim of the policy of health regionalization in hospital care would be to assure access and reduce inequalities, which, in the state of Sao Paulo, is an unfinished task. Table 4 could be complemented with the study of health problems – diagnoses and treatments – that constitute specific demands in the different regions of the state.

The public and private hospitalization systems are not stagnant. There are exchanges between them, depending on the oscillation of the funding of public care and private plans. Thus, this view applies to the year of the study, and the future trends of the flows will follow the policies of hospital care.

## Footnotes

a Ministério da Saúde. Portaria nº 399, de 22 de fevereiro de 2006. Divulga o Pacto pela Saúde 2006 − Consolidação do SUS e aprova as Diretrizes Operacionais do Referido Pacto. Brasília (DF); 2006 [cited 2014 Feb 24]. Available from: http://bvsms.saude.gov.br/bvs/saudelegis/gm/2006/prt0399_22_02_2006.html

b Ministério da Saúde. Portaria nº 699, de 30 de março de 2006. Regulamenta as Diretrizes Operacionais dos Pactos Pela Vida e de Gestão. Brasília (DF); 2006 [cited 2014 Feb 24]. Available from: http://dab.saude.gov.br/docs/legislacao/portaria699_30_03_06.pdf html

c Ministério da Saúde. Portaria nº 4.279, de 30 de dezembro de 2010. Estabelece diretrizes para a organização da Rede de Atenção à Saúde no âmbito do Sistema Único de Saúde (SUS). Brasília (DF); 2010 [cited 2014 Apr 17]. Available from: http://bvsms.saude.gov.br/bvs/saudelegis/gm/2010/prt4279_30_12_2010.html

d Moreira ML. Readmissões no sistema de serviços hospitalares no Brasil [thesis]. São Paulo (SP): Faculdade de Medicina da USP; 2010.
