# Supplementary material for: Hospitalization flow in the public and private systems in the state of Sao Paulo, Brazil
Source: Rev Saude Publica. 2015 Oct 2;49:69. doi: 10.1590/S0034-8910.2015049005696 (PMC4593328; doi:10.1590/S0034-8910.2015049005696)
Supplement: Translation

# Translation: Fluxo de hospitalização nos sistemas público e privado no estado de São Paulo

**Authors:** Juan Stuardo Yazlle Rocha, Rosane Aparecida Monteiro, Marizélia Leão Moreira

**Affiliations:** I Departamento de Medicina Social. Faculdade de Medicina de Ribeirão Preto. Universidade de São Paulo. Ribeirão Preto, SP, Brasil; II Agência Nacional de Saúde Suplementar. Rio de Janeiro, RJ, Brasil

**Keywords:** Hospitalização, Hospitais Municipais, provisão & distribuição, Migração, Regionalização, Equidade no Acesso

## Abstract

**OBJETIVO:**

Descrever os fluxos migratórios de demanda por assistência hospitalar pública e privada entre as regiões de saúde no estado de São Paulo, Brasil.

**MÉTODOS:**

Estudo com base em banco de dados de hospitalizações nos sistemas público e privado, no estado de São Paulo, em 2006. Foram analisados dados de 17 regiões de saúde do estado, considerando aqueles hospitalizados na própria região de saúde e os que migraram para fora (evasão) ou vieram de outras regiões (invasão). Calculou-se o índice de eficácia migratória de pacientes de ambos os sistemas. Foi analisada a cobertura (coeficiente de hospitalização) em relação ao número de leitos por habitantes e os índices de eficácia migratória.

**RESULTADOS:**

O índice de eficácia migratória aplicado ao fluxo de demanda da assistência hospitalar permitiu caracterizar as regiões de saúde com equilíbrio de fluxos, com alta evasão de pacientes públicos e privados, e com alta atração de pacientes públicos e privados.

**CONCLUSÕES:**

Há diferenças de acesso e oportunidades à assistência hospitalar entre as regiões de saúde no estado de São Paulo.

## INTRODUÇÃO

De acordo com Viana (2011),^10^ a política social e de saúde no Brasil pode ser dividida em três períodos. O período da institucionalidade liberal (1995-2002) é caracterizado pela ênfase na desregulamentação e autorregulação dos mercados com a criação das Agências reguladoras da Saúde Suplementar (ANS) e de Vigilância Sanitária (ANVISA) e mais a estratégia chave da descentralização com ênfase na atenção primária. A fase de transição (2003-2006), com saldos positivos tanto em sua balança comercial quanto na de pagamentos, desenvolveu o programa emblemático da bolsa família. A fase da institucionalidade neodesenvolvimentista (2007-2010) é caracterizada pela emergência da nova classe média, que concentra grande poder de compra, e a regionalização na saúde, aliada à forte expansão dos investimentos voltados à construção de equipamentos de saúde – ambulatórios e hospitais –, assim como ao estímulo ao fortalecimento do complexo econômico-industrial da saúde.

Estudos do desenvolvimento social e econômico voltam-se para o papel do setor saúde. Este, além dos serviços disponíveis, mobiliza a base industrial: química, biotecnológica e de mecânica, eletrônica e de materiais. O setor de serviços possui o maior peso do complexo econômico industrial da saúde.^2^ Sua organização segue a dos espaços e conforma pólos e regiões.

O planejamento da organização da assistência em níveis regionais é recente no Brasil. Embora enunciado na prescrição constitucional do Sistema Único de Saúde (SUS) de 1988, só avançou com a edição das Normas Operacionais de Assistência à Saúde (NOAS), de 2001 e 2002. A NOAS 2002 estimulou a criação de Planos Diretores de Regionalização e a organização de redes de assistência em bases regionais conforme as necessidades da população, segundo parâmetros epidemiológicos – e não pela existência e oferta de serviços.

Em 2006, a regionalização e hierarquização dos serviços de saúde, notadamente os de alta complexidade, aparecem nas diretrizes políticas e organizacionais emanadas do Pacto de Gestão, (Portaria MS 399)^a^ no Regulamento e nas Diretrizes dos Pactos pela Vida e de Gestão (Portaria MS 699),^b^ e nas Redes de Atenção à Saúde.^c^

Oliveira et al^6^ (2008) estudaram a atenção à saúde pelo SUS no estado de São Paulo, com foco na perspectiva regional no contexto da elaboração do Plano Estadual de Saúde. Foram utilizados dados do Sistema de Informação Hospitalar (SIH) de 2000 e 2006 para a assistência hospitalar e da ANS para estimar a população coberta por planos de saúde. A esse respeito, afirmam:

“Os gestores da saúde estão diante de um impasse: considerar ou não a cobertura dos planos de saúde no processo de planejamento do SUS? Com o crescimento da população coberta e a disparidade dessa cobertura entre as regiões e, sobretudo entre os municípios, a não consideração desse fator pode contribuir para a manutenção das desigualdades de acesso atualmente identificadas no sistema”.

“No Estado de São Paulo, os leitos SUS cadastrados no Cadastro Nacional de Estabelecimentos de Saúde (CNES) são na maioria filantrópicos (46,9% dos leitos); 40,2% são públicos e 12,9% são leitos privados, contratados ao SUS. Esta característica exige grande cuidado na política de parceria com os hospitais filantrópicos, sob pena de colocar em risco o atendimento de usuários do SUS, em algumas regiões onde há maior dependência destes leitos hospitalares”.

A regionalização é problematizada por Machado.^4^ A criação de redes de assistência seria a garantia do atendimento equânime e integral a todos os cidadãos brasileiros – supondo a articulação entre municípios “exportadores” e “importadores” de serviços, sob a coordenação dos governos estaduais. Todavia, a disposição para a cooperação estaria longe de se concretizar. Venâncio et al^9^ analisaram as práticas gestoras de referência regional em cinco regiões do estado de São Paulo, avaliando indicadores, instrumentos utilizados e a percepção dos gestores regionais e municipais. Os mecanismos formais de referência regional foram considerados insuficientes, bem como os instrumentos para seu acompanhamento.

A regionalização da saúde estava presente como preocupação de dirigentes de saúde há muitos anos,^1^ em torno da articulação entre municípios exportadores e importadores de pacientes. A lógica presumida do fluxo é que quanto maior a evasão de pacientes, menor seria a capacidade de assistir local ou regionalmente a população. Por outro lado, quanto maior a invasão de pacientes, maior o poder de atração exercido pelas condições de oferta de assistência. Cada região possui um município pólo que concentra os recursos de assistência de nível secundário e terciário e receberia o fluxo total da sua área de referência. Além disso, existem recursos diagnósticos e de tratamento de nível intermediário em outros municípios satélites do pólo regional. Municípios e regiões importadores são também, em alguma medida, exportadores. Deve-se elucidar o predomínio de um ou outro tipo de fluxo. Para isso, é preciso considerar o tamanho da população de referência, a intensidade do fluxo em um ou outro sentido e a soma de ambos os fluxos em relação ao total da demanda no período.

O estudo do fluxo das hospitalizações em serviços públicos e privados pode contribuir para o conhecimento das necessidades e demandas populacionais na saúde em bases regionalizadas e a adequação da estrutura das regiões e comparações entre elas. Contar com um instrumento objetivo de avaliação da intensidade e sentido dos fluxos é importante para a estruturação regional da assistência à saúde. A procura de recursos diagnósticos e de tratamento fora do local de residência permite inferir o grau de resolutividade da assistência segundo os módulos assistenciais vigentes. Embora a universalidade do SUS estabeleça o direito à assistência em qualquer serviço do sistema, é importante conhecer a procedência dos pacientes (município de residência) para o planejamento da assistência à demanda local e regional. Conceitos como importação e exportação, referência para fora ou referência para dentro e invasão ou evasão de pacientes no sentido dos conceitos demográficos de imigração ou emigração são utilizados com certa impropriedade na literatura. Utilizamos neste trabalho invasão (imigração) e evasão (emigração) pelo uso consagrado em estudos epidemiológicos.

Este estudo teve por objetivo descrever os fluxos de demanda por assistência hospitalar pública e privada entre as regiões de saúde no Estado de São Paulo.

## MÉTODOS

Estudo exploratório, com base em recorte do banco de dados construído por Moreira^d^ contendo os registros de todas as hospitalizações no Brasil pelos sistemas público e privado (planos privados e particulares) em 2006. O estudo de Moreira tinha como objetivo analisar as reinternações no Brasil com dados dos sistemas Sistema de Internações Hospitalares (SIH)/SUS – e Comunicações de Internações Hospitalares (CIH) do Datasus. Foi criado um banco de dados a partir de sofisticado processo de associação de registros (*Reclink*) e *linkage* com as variáveis nome, sexo, município de residência e data de nascimento, mediante algoritmos previamente definidos e testados. Como no SIH, cada evento (hospitalização) pode corresponder a uma cobrança parcial, de parte do período ou de alguns procedimentos da internação – situação também aceita nas CIH do período – foram definidos três algoritmos para identificar os eventos da mesma internação e compor o registro de seus dados. Assim, foi possível identificar as internações de pacientes pagas pelo SUS e por sistema privado, bem como as suas repetições informadas ao Ministério da Saúde pelo SIH e CIH, respectivamente.^5^

Para o presente estudo, foram selecionadas todas as internações financiadas pelo SUS e sistema privado de pessoas residentes no estado de São Paulo, incluindo as que ocorreram em outros estados da União, de 1 de janeiro a 31 de dezembro de 2006. As hospitalizações foram classificadas segundo o município de residência e sua localização em uma das 17 regiões de saúde do estado. A população estimada, o número de leitos gerais e o índice de leitos por mil habitantes de cada Departamento Regional de Saúde (DRS), em 2006, foram obtidos do Plano Estadual de Saúde.^8^ As internações foram classificadas segundo cada DRS (total de internações) e tabuladas como internações de pacientes residentes na mesma região ou não, de acordo com o município de origem do paciente. Isso permitiu calcular o número e percentual de pacientes atendidos na própria região; o total de internações em cada região (coluna C, Tabelas 1 e 2) menos o total de internados na própria origem, (coluna E, Tabelas 1 e 2) que fornece os casos de invasão, ou seja, o número de casos vindos de outras regiões; o total de residentes internados (coluna D, Tabelas 1 e 2) menos o total de internados na região de origem (coluna E, Tabelas 1 e 2), fornece o número de casos de evasão, em que a hospitalização ocorreu fora da própria região do paciente. O total de internados de cada DRS, em relação à população residente por mil habitantes, mostra o coeficiente de hospitalização total de cada uma das regiões do Estado.

A intensidade da invasão e evasão de pacientes foi calculada pelo indicador de eficácia migratória (EM), utilizado por Caiado.^2^ Esse indicador permite quantificar a intensidade dos fluxos de entrada e de saída e o sentido predominante. Ele é calculado pelo quociente entre a migração liquida (Invasão menos a Evasão) e a migração bruta (Evasão mais Invasão).



Valores próximos a 1 indicam forte atração migratória, próximos a -1 indicam áreas de alta evasão de casos, e próximos a 0, áreas com alta circulação migratória.

Foram calculados os coeficientes de correlação entre o índice de leitos hospitalares e o coeficiente de hospitalizações por mil habitantes. Foi analisado o índice de eficácia migratória de invasão e evasão de pacientes dos sistemas público (SUS) e privados (NÃO-SUS) em cada Departamento Regional de Saúde.

## RESULTADOS

Houve 2.922.648 hospitalizações no estado de São Paulo em 2006, das quais 2.808.765 eram de residentes em São Paulo internados no próprio estado e 83.389 de internados em outros estados, totalizando 2.892.154 internações de residentes em São Paulo (Tabela 3). A população dos municípios pólo é de pouco mais de 15 milhões, o que significa que a maioria das regiões possui uma população em área de referência satélite maior que a residente nos pólos – quase 10 vezes maior em Taubaté e São João da Boa Vista e pouco menos do dobro na Grande São Paulo. Os fluxos de demanda de serviços resultantes representaram a dinâmica da regionalização no estado.

**Tabela 3:** Distribuição da população, internações, índice leitos e coeficiente hospitalizações, segundo as regiões de saúde. Estado de São Paulo, 2006.

Regionais de Saúde	População	População no município pólo	% População no pólo	Total de internações de residentes	Índice de leitos/1.000 habitantes	Coeficiente de hospitalização por mil habitantes (residência/população)
3501 Grande São Paulo	19.677.510	10.603.309	53,9	1.168.968	1,36	59,41
3502 Araçatuba	700.008	174.770	25,0	55.645	2,12	79,49
3503 Araraquara	915.240	192.153	21,0	74.223	1,52	81,1
3504 Baixada Santista	1.666.453	402.664	24,2	116.692	1,46	70,02
3505 Barretos	412.722	106.048	25,7	43.081	2,55	104,38
3506 Bauru	1.623.025	343.285	21,1	153.389	2,45	94,51
3507 Campinas	3.885.612	1.019.655	26,2	273.450	1,19	70,38
3508 Franca	657.344	315.795	48,0	55.075	1,57	83,78
3509 Marília	1.081.290	215.673	19,9	111.738	2,36	103,34
3510 Piracicaba	1.405.849	352.684	25,1	85.089	1,31	60,52
3511 Presidente Prudente	723.244	198.937	27,5	80.297	2,41	111,02
3512 Registro	299.360	55.141	18,4	13.462	1,03	44,97
3513 Ribeirão Preto	1.261.413	538.639	42,7	88.713	1,82	70,33
3514 São João da Boa Vista	797.952	80.334	10,1	83.692	2,05	104,88
3515 São José do Rio Preto	1.459.320	399.904	27,4	186.250	2,49	127,63
3516 Sorocaba	2.245.623	556.366	24,8	168.920	1,24	75,22
3517 Taubaté	2.243.796	261.454	11,6	133.470	1,42	59,48
Total	41.055.761	15.816.811	38,5	2.892.154	1,53	70,44

O índice geral de leitos por habitantes do estado foi baixo (1,53 por mil habitantes), com grande variação entre as regiões (Tabela 3). Os Departamentos Regionais de Saúde que apresentaram maior índice de leitos por habitantes foram Barretos (2,6), São José do Rio Preto (2,5), Bauru (2,5) e Presidente Prudente (2,4). Barretos e Bauru possuem hospitais especializados que são referências nacionais nas áreas de oncologia e defeitos da face, o que explica o elevado índice de leitos ali existentes. Os Departamentos Regionais com menores índices de leitos por habitantes foram Registro (1,0), Campinas (1,2) e Sorocaba (1,2). O coeficiente geral de hospitalizações, incluindo internações públicas e privadas (SUS e sistema particular) do estado de São Paulo foi baixo (70,4 internações por mil habitantes), mas com grande variação entre as regiões. As regiões de saúde com os maiores coeficientes de internações por mil habitantes foram São José do Rio Preto (127,6), Presidente Prudente (111,0), São João da Boa Vista (104,9), Barretos (104,4) e Marília (103,3). Aquelas com menores coeficientes de hospitalizações da população residente foram Registro (45,0), Grande São Paulo (59,4), Taubaté (59,5) e Piracicaba (60,5). O cálculo do coeficiente de correlação indicou elevada associação positiva entre o índice leitos por habitantes e o coeficiente de internações por mil habitantes (r = 0,88). Na média estadual, 69,9% das internações no estado ocorreram pelo SUS e os maiores percentuais ocorreram nas regiões de Registro (95,8), Araçatuba (86,8) e Taubaté (81,0) e as menores em Presidente Prudente (61,4), Campinas (62,2) e Franca (64,2) (Tabela 1). No geral, as internações pelo SUS ocorreram na própria região em 97,6% dos casos – sugerindo a adequação da regionalização do SUS, i.e., 2,4% procuraram assistência com sucesso em outra região (evasão). As regiões de saúde atenderam 3,2% de casos provenientes de outras regiões do estado (invasão). Todas as regiões de saúde tiveram casos de invasão e evasão (colunas F e G). O cálculo da eficácia migratória (coluna H) permitiu quantificar e detectar o predomínio do fluxo em um ou em outro sentido. Foram considerados os valores positivos acima de 0,50 como indício de forte atração (invasão) migratória pelo SUS, caso das regiões de Bauru (0,78), Ribeirão Preto (0,69), Barretos (0,65) e Grande São Paulo (0,56), i.e., regiões que atraem fluxos de pacientes por assistência pelo SUS. Inversamente, as regiões que apresentaram índice de eficácia migratória negativa (abaixo de -0,50) foram consideradas como forte evasão do SUS, caso das regiões de Franca (-0,80), Marília, Araraquara e Registro (-0,77) e Araçatuba (-0,71), i.e., regiões que estimulam a emigração e não oferecem cobertura local da demanda, embora Araçatuba e Marília tenham elevados índice de leitos/habitantes.

**Tabela 1:** Total de internações pelo Sistema Único de Saúde (SUS) segundo o local de atendimento, total de internações de residentes, internações no local de origem, invasão e evasão (número e coeficiente) e índice de eficácia migratória. Estado de São Paulo, 2006.

Regionais de saúde	Internações SUS por local de atendimento (C)		Total de internações de residentes (D)	Internações no local de origem (E)		Casos de invasão (F)		Casos de evasão (G)		Índice de eficiência migratória (H)
	n	%		n	%	n	%	n	%	
3501 Grande São Paulo	834.367	70,5	821.626	816.707	99,4	17.660	2,1	4.919	0,6	0,56
3502 Araçatuba	44.840	86,8	47.117	44.370	94,2	470	1,0	2.747	5,8	-0,71
3503 Araraquara	47.057	66,5	49.966	46.612	93,3	445	0,9	3.354	6,7	-0,77
3504 Baixada Santista	75.347	66,2	77.121	74.569	96,7	778	1,0	2.552	3,3	-0,53
3505 Barretos	35.205	74,7	29.531	27.992	94,8	7.213	20,5	1.539	5,2	0,65
3506 Bauru	124.326	75,0	112.994	111.427	98,6	12.899	10,4	1.567	1,4	0,78
3507 Campinas	179.028	62,2	174.807	170.262	97,4	8.766	4,9	4.545	2,6	0,32
3508 Franca	34.050	64,2	36.016	33.810	93,9	240	0,7	2.206	6,1	-0,80
3509 Marília	78.919	72,9	82.005	78.466	95,7	453	0,6	3.539	4,3	-0,77
3510 Piracicaba	59.262	72,0	62.199	58.298	93,7	964	1,6	3.901	6,3	-0,60
3511 Presidente Prudente	49.215	61,4	50.122	48.915	97,6	300	0,6	1.207	2,4	-0,60
3512 Registro	11.729	95,8	12.731	11.579	91,0	150	1,3	1.152	9,0	-0,77
3513 Ribeirão Preto	71.734	76,4	67.247	66.220	98,5	5.514	7,7	1.027	1,5	0,69
3514 São João da Boa Vista	53.505	66,1	56.639	52.449	92,6	1.056	2,0	4.190	7,4	-0,60
3515 São José do Rio Preto	113.262	58,3	111.737	109.302	97,8	3.960	3,5	2.435	2,2	0,24
3516 Sorocaba	123.793	74,4	125.766	121.252	96,4	2.541	2,1	4.514	3,6	-0,28
3517 Taubaté	106.506	81,0	107.498	104.545	97,3	1.961	1,8	2.953	2,7	-0,20
Total	2.042.145	69,9	2.025.122	1.976.775	97,6	65.370	3,2	48.347	2,4	0,15

Na média estadual, 30,1% das internações ocorreram por sistemas privados, as mais elevadas nas regiões de São José do Rio Preto (41,7), Presidente Prudente (38,6), Campinas (37,8) e Franca (35,8); as menores ocorreram em Registro (4,2), Araçatuba (13,2), Taubaté (19,0) (Tabela 2). O cálculo do índice de eficácia migratória apontou as regiões de São José do Rio Preto (0,87) e Campinas (0,64) como aquelas que exercem maior atração de internações por planos privados. A emigração (evasão) à procura de assistência privada foi mais intensa em Registro (-0,87), região com menor índice de leitos habitantes, Araçatuba (-0,81), Barretos (-0,70) e Baixada Santista (-0,56).

**Tabela 2:** Distribuição das internações totais financiadas por sistemas privados, invasão e evasão (número e coeficiente) e índice de eficácia migratória. Estado de São Paulo, 2006.

Regionais de saúde	Total de internações por sistemas privados por local de atendimento (C)		Total de internações de residentes (D)	Internações no local de origem (E)		Casos de invasão (F)		Casos de evasão (G)		Índice de eficácia migratória (H)
	n	%		n	%	n	%	n	%	
3501 Grande São Paulo	348.423	29,5	347.342	335.934	96,7	12.489	3,6	11.408	3,3	0,05
3502 Araçatuba	6.815	13,2	8.528	6.615	77,6	200	2,9	1.913	22,4	-0,81
3503 Araraquara	23.690	33,5	24.257	22.405	92,4	1.285	5,4	1.852	7,6	-0,18
3504 Baixada Santista	38.454	33,8	39.571	38.013	96,1	441	1,1	1.558	3,9	-0,56
3505 Barretos	11.923	25,3	13.550	11.571	85,4	352	3,0	1.979	14,6	-0,70
3506 Bauru	41.401	25,0	40.395	38.957	96,4	2.444	5,9	1.438	3,6	0,26
3507 Campinas	108.733	37,8	98.643	95.863	97,2	12.870	11,8	2.780	2,8	0,64
3508 Franca	18.973	35,8	19.059	18.345	96,3	628	3,3	714	3,7	-0,06
3509 Marília	29.272	27,1	29.733	27.832	93,6	1.440	4,9	1.901	6,4	-0,14
3510 Piracicaba	23.077	28,0	22.890	20.918	91,4	2.159	9,4	1.972	8,6	0,05
3511 Presidente Prudente	30.964	38,6	30.175	29.330	97,2	1.634	5,3	845	2,8	0,32
3512 Registro	519	4,2	731	503	68,8	16	3,1	228	31,2	-0,87
3513 Ribeirão Preto	22.181	23,6	21.466	20.861	97,2	1.320	6,0	605	2,8	0,37
3514 São João da Boa Vista	27.390	33,9	27.053	25.390	93,9	2.000	7,3	1.663	6,1	0,09
3515 São José do Rio Preto	81.149	41,7	74.513	74.033	99,4	7.116	8,8	480	0,6	0,87
3516 Sorocaba	42.569	25,6	43.154	41.256	95,6	1.313	3,1	1.898	4,4	-0,18
3517 Taubaté	24.970	19,0	25.972	24.164	93,0	806	3,2	1.808	7,0	-0,38
Total	880.503	30,1	867.032	831.990	96,0	48.513	5,5	35.042	4,0	0,16

O estudo dos fluxos da demanda de assistência hospitalar no estado de São Paulo em 2006 apontou como regiões problemáticas Araçatuba, Registro e Baixada Santista, que apresentaram forte emigração (evasão) de pacientes para a atenção pública e para a privada, configurando um quadro de carência de assistência pública e privada (Tabela 4). As regiões de Araraquara, Franca, Marília, Piracicaba, Presidente Prudente e São João da Boa Vista apresentaram elevado índice migratório para assistência pública. Duas regiões – Campinas e São José do Rio Preto – tiveram elevada atração por assistência privada, e ambas apresentaram equilíbrio nos fluxos do SUS. A região de Ribeirão Preto teve fluxo público elevado e equilíbrio nos fluxos privados. As regiões de Sorocaba e Taubaté apresentaram equilíbrio nos fluxos público e privado.

**Tabela 4:** Caracterização das Regiões de Saúde do estado de São Paulo, segundo os fluxos por assistência hospitalar pública e privada. Estado de São Paulo, 2006.

Regionais de Saúde	Equilíbrio de fluxos		Forte atração de fluxos		Forte evasão de fluxos	
	Público	Privado	Público	Privado	Público	Privado
Grande São Paulo		X	X			
Araçatuba					X	X
Araraquara		X			X	
Baixada Santista					X	X
Barretos			X			X
Bauru		X	X			
Campinas	X			X		
Franca		X			X	
Marília		X			X	
Piracicaba		X			X	
Presidente Prudente		X			X	
Registro					X	X
Ribeirão Preto		X	X			
São João Boa Vista		X			X	
São José do Rio Preto	X			X		
Sorocaba	X	X				
Taubaté	X	X				

## DISCUSSÃO

Os municípios do estado de São Paulo apresentavam grandes diferenças quanto ao desenvolvimento socioeconômico e densidade populacional em 2009,^7^ 11,3% deles possuíam população acima de 100 mil habitantes e todos eram classificados como de alta riqueza no Índice Paulista de Responsabilidade Social; 61,7% possuíam menos de 20 mil habitantes e 70,4% eram classificados na categoria de baixa riqueza. Embora apenas 73 municípios estivessem incluídos entre os grandes, eles reuniam quase 75,0% da população do estado. Isso ilustra a grande dependência dos municípios menores em relação aos maiores e, portanto, a importância da questão da regionalização da saúde no estado de São Paulo. Municípios maiores, mais ricos, se tornam pólos e atraem a população dos municípios menores, mais pobres. Assim, a regionalização é o instrumento para tentar compensar a desigualdade e propiciar igualdade de oportunidades às populações menos favorecidas.

A elaboração do indicador de eficácia de migração, aplicado ao estudo dos fluxos da demanda de hospitalizações no estado de São Paulo, resultou em um índice que permite o ordenamento quantitativo da proporção de casos de invasão e evasão de pacientes, entre as regiões de saúde do estado, e da prevalência maior de uma ou outra. Essa visão facilita a classificação qualitativa dos municípios e regiões de saúde segundo apresentem equilíbrio nos fluxos migratórios ou exerçam forte atração ou evasão de pacientes pela assistência pública (SUS) ou privada. Foi surpreendente encontrar que a região da Grande São Paulo – concentradora de recursos hospitalares, especialmente de nível terciário, mas com grande concentração populacional – apresentou baixo coeficiente de hospitalizações da população local, sugerindo possível carência relativa de leitos gerais por demanda externa muito maior. As regiões de São José do Rio Preto e de Campinas não apresentaram evasão de pacientes para assistência SUS – a primeira com elevado índice de leitos habitantes e a segunda não. A região de Registro apresentou elevada evasão por demanda de assistência hospitalar privada, provavelmente por falta de oferta de assistência pública. A região de Araçatuba, com 2,12 leitos por mil habitantes, apresentou elevada evasão por assistência privada, provavelmente por problemas de acesso aos leitos existentes.

Embora o total de casos detectados como de invasão ou evasão seja relativamente pequeno (5,6% e 9,5% das internações públicas e privadas), ele aflige uma população fragilizada pela doença. A política de regionalização da saúde na assistência hospitalar teria por objetivo garantir o acesso e diminuir iniquidades o que, no estado de São Paulo, é uma tarefa não concluída. A Tabela 4 poderia ser complementada com o estudo dos problemas de saúde – diagnósticos e tratamentos – que caracterizam demandas específicas nas diferentes regiões do estado.

Os sistemas público e privado de hospitalizações não são estanques. Há trocas entre eles a depender da oscilação do financiamento da assistência pública e dos planos privados. Assim, o quadro vale para o ano do estudo e as tendências futuras dos fluxos seguirão as políticas da assistência hospitalar.

## Footnotes

a Ministério da Saúde. Portaria nº 399, de 22 de fevereiro de 2006. Divulga o Pacto pela Saúde 2006 − Consolidação do SUS e aprova as Diretrizes Operacionais do Referido Pacto. Brasília (DF); 2006 [citado 2014 fev 24]. Disponível em: http://bvsms.saude.gov.br/bvs/saudelegis/gm/2006/prt0399_22_02_2006.html

b Ministério da Saúde. Portaria nº 699, de 30 de março de 2006. Regulamenta as Diretrizes Operacionais dos Pactos Pela Vida e de Gestão. Brasília (DF); 2006 [citado 2014 fev 24]. Disponível em: http://dab.saude.gov.br/docs/legislacao/portaria699_30_03_06.pdf html

c Ministério da Saúde. Portaria nº 4.279, de 30 de dezembro de 2010. Estabelece diretrizes para a organização da Rede de Atenção à Saúde no âmbito do Sistema Único de Saúde (SUS). Brasília (DF); 2010 [citado 2014 abr 17]. Disponível em: http://bvsms.saude.gov.br/bvs/saudelegis/gm/2010/prt4279_30_12_2010.html

d Moreira ML. Readmissões no sistema de serviços hospitalares no Brasil [tese]. São Paulo (SP): Faculdade de Medicina da USP; 2010.
